# From digital voices to vaccine choices: increasing female vaccine acceptance in Sudan through social listening

**DOI:** 10.3389/fgwh.2024.1288559

**Published:** 2024-02-12

**Authors:** Islam Ahmed, Hiba Ali, Sehrish Ali, Simon Van Woerden, Antonio Hanna-Amodio, Katie Chen, Elizabeth Onitolo, Amaya Gillespie

**Affiliations:** ^1^ UNICEF Sudan, Social and Behaviour Change, Khartoum, Sudan; ^2^ The Behavioural Insights Team, London, United Kingdom; ^3^ Maiduguri Field Office, UNICEF Nigeria, Maiduguri, Nigeria; ^4^ UNICEF Middle East and North Africa Regional Office, Social and Behaviour Change, Amman, Jordan

**Keywords:** vaccine acceptance, women, social listening, Sudan, COVID-19

## Abstract

The early COVID-19 vaccine rollout in Sudan experienced a gender disparity in vaccine uptake, with women accounting for less than 40% of vaccinations after four months of vaccine access. Initial analysis revealed that demand generation approaches were not sufficiently tailored to address the challenges and concerns of women. Using real-time social media monitoring, also known as social listening, to understand this inequity, we used an analytical tool called “Talkwalker” to track public sentiment and engagement regarding vaccination on social media platforms. The data captured subsequently informed a gender-responsive messaging campaign on social media that directly addressed specific concerns of Sudanese women. Within one week of the campaign's onset in September 2021, we observed a 144% increase in women's COVID-19 related social media engagement. Subsequent campaigns further enhanced women's engagement from 31% to between 35%–47%. Two subsequent campaigns in January and February/March 2022 were complemented with in-person activities, such as group meetings with community members and home visits by health promoters. Women's vaccination rates increased by 9% while the first two campaigns were live. System constraints hindered data linkages for the third campaign. These findings demonstrate the value of integrating real-time feedback with large-scale social media campaigns and the potential of linking online and offline strategies to further refine interventions, particularly in a conflict-prone and low-income country context. Our experience reinforces the notion that “one size does not fit all” when it comes to health-related communication. Responses should be tailored, contextualized, and person-centered, particularly in addressing concerns unique to women.

## 1 Introduction

The Corona virus disease (COVID-19) pandemic necessitated rapid, large-scale vaccination efforts worldwide to slow the virus's spread and attenuate its impact. However, in many countries, equitable vaccine distribution presented a significant challenge (1). Such disparities emerged in Sudan, the first country in the Middle East and North Africa (MENA) region to receive COVID-19 vaccines through the COVAX initiative (2). Despite concerted efforts to broaden coverage, vaccine equity persisted as a significant issue: with women accounting for less than 40% of the vaccinated population by the end of 2021 (3). A comparative analysis of the first two government-led vaccination drives revealed that not only were women less likely to get vaccinated, but their engagement with COVID-19 related discourse on social media platforms was also consistently low (4). It is estimated that only around 30% of the Sudanese population have internet access, and women are reported to have less access than men (5). Nevertheless, social media cannot be overlooked as a core part of programming, and adjunct to off-line qualitative and quantitative data, including epidemiological data.

From the onset of the COVID-19 pandemic, the Federal Ministry of Health led continuous campaigns adapting key messages to the *constantly* evolving situation. Uncertainty and the rapid pace of change, as well as the global shortage of vaccines, fed into widespread misinformation in Sudan virtually from the outset, as was the case around the world. Campaigns tended to focus on the science and expert opinion from early 2020, with reference to understanding symptoms and transmission, as well as preventive measures, and increasingly included the potential of vaccines towards the end of 2020, and direct vaccination promotion as vaccines became available in the region during 2021. Simply conveying the scientific facts proved increasingly inadequate as the pandemic evolved, and public trust fluctuated. Traditional data collection, such as randomised controlled trials or nationally representative studies, also proved unable to keep pace with changing community demands and context. Against this backdrop, social listening was introduced as a way to monitor community sentiment and concerns in real time, to facilitate rapid adaptation of COVID-19 programming.

Gender inequity in vaccination uptake has been observed across countries, age-groups and vaccine type (6–8). The underlying causes of this gender disparity are likely to be multi-layered and linked with societal norms, misinformation, and logistical challenges (9). In Sudan during the COVID-19 vaccine rollout, vaccinators and programme managers on the ground anecdotally reported that one of the possible reasons for higher coverage for men was that, in many cases, vaccination was required to travel overseas for employment in countries such as Saudi Arabia, as well as required for employment within the same country. Similarly, there was also a common perception that because men were out of the house more often, they needed to be protected, while alternatively as women spent most of their time at home, they would be safer. Additionally, concerns about the vaccine resulting in infertility and a lack of opportunity for privacy with female vaccinators, were also reported as contributors to lower vaccine uptake among women in Sudan. Initial public health communication, in short, did not sufficiently differentiate concerns and considerations specific to Sudanese women.

Gaining a deeper understanding of these specific gender barriers helped in informing a strategic shift in public health messaging and community engagement, to encourage vaccine uptake. This paper presents a summary of an initiative led by UNICEF in partnership with the Federal Ministry of Health in Sudan that applied “Talkwalker”, a real-time social media monitoring and analytical tool, to address this gender disparity and encourage uptake of COVID-19 vaccinations by women.

## 2 Method

Social listening refers to both online and offline activities. In online settings, it involves tracking public conversations on social media platforms to gauge and track changes in public sentiment towards a particular issue (10). Although the focus of this paper is the online component, offline feedback was also gathered, by leveraging community mobilizers, field-based project staff, and service providers to engage in direct communication with populations of interest (11). In this context, the aim was to understand the barriers to vaccine uptake and the reasons driving low vaccine acceptance, in particular among women.

Data obtained for the purpose of this research were obtained from open source social media platforms such as Facebook and Twitter, and no individual identifiers were collected. Given that all online data were public, no specific ethical approvals were required. Furthermore the names and account pictures of comments quoted in this paper on the gender responsive campaign were concealed, thus keeping the identity of these individuals anonymous. All offline data were collected from official staff and sources as part of routine work, and also kept anonymous, under the auspices of the Federal Ministry of Health.

In collaboration with the government, UNICEF began employing social listening in August 2021, as a monitoring tool to track general responses to COVID-19 and, in particular, to monitor social media conversations concerning vaccines. The aim was to produce timely data to inform vaccine demand and service delivery. However, gender disparities became evident early in the process. For instance, rumor tracking dashboards, frequently asked questions on the Ministry of Health website and feedback from field-based teams, suggested that women had particular concerns about their fertility, the health of their unborn babies, and the safety of the vaccine during menstruation, pregnancy, and lactation.

Insights from social listening were fed back to the risk communication and community engagement (RCCE) sub-committee under the COVID-19 National Technical Committee on a weekly basis at the federal level, and cascaded to state sub-committees*.* Notably, data gleaned from social listening was integrated into health promotion capacity-building prior to the campaign design and rollout. Social listening identified gender-specific concerns, which supported the development of Frequently Asked Questions (FAQ's), mobilizing the relevant influencers and designing female-friendly content, ultimately leading to increased two-way engagement. Inviting female audiences to tell their stories or ask questions was imperative in fostering a sense of trust, authenticity, and credibility.

**Figure 1:**
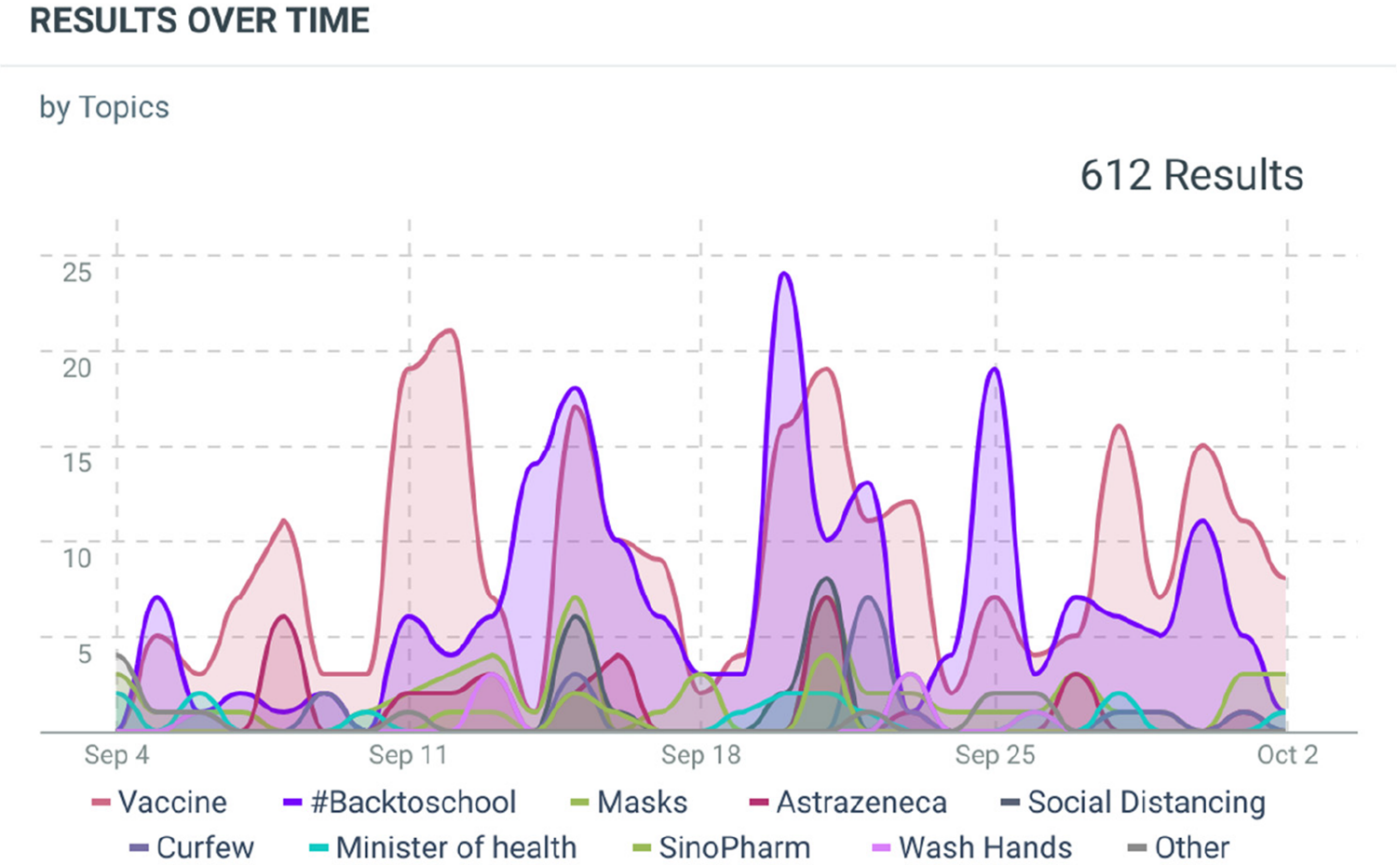
Extract from Talkwalker dashboard showing volume of conversations on COVID-19 related topics from main social media platforms (Facebook, Twitter, online news websites) between 4th September and 2nd October, 2021.

**Figure 2:**
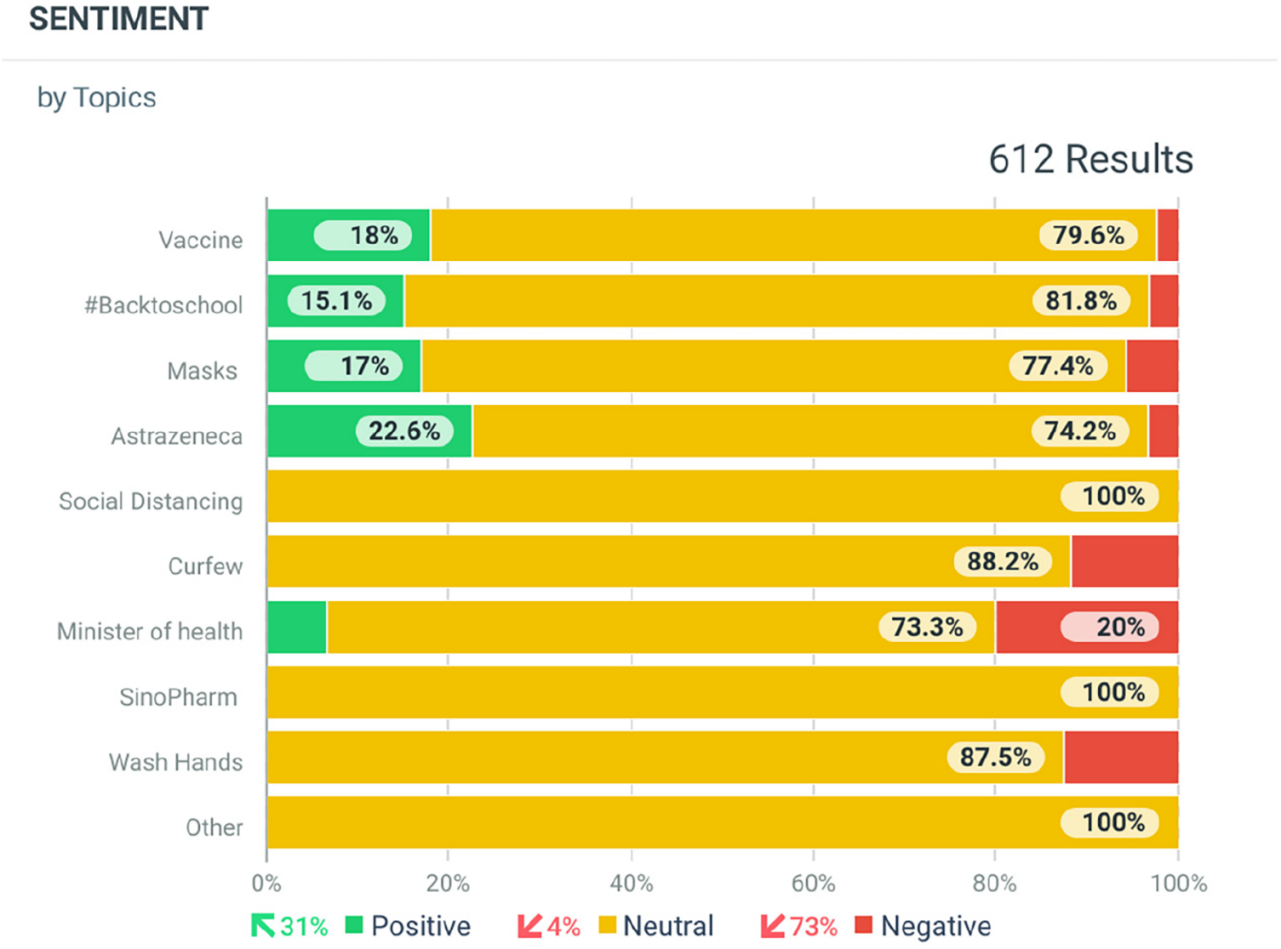
Extract from Talkwalker dashboard showing sentiment analysis for COVID-19 related topics from main social media platforms (Facebook, Twitter, online news websites) between 4th September and 2nd October, 2021.

The Talkwalker application was the social listening tool used by UNICEF Sudan. The application monitored keywords and topics mentioned in social media feeds related to COVID-19. For example, data is analysed through tracking keywords, such as vaccine, COVID-19, handwashing, and face masks. The Talkwalker application was programmed to produce graphs highlighting the number and type of COVID-19 conversations in Sudan as shown in Figure 1. Furthermore analysis of sentiments towards COVID-19 on social media is shown in Figure 2. The resulting dashboard, which was monitored on a weekly basis, became a valuable resource for structuring vaccine promotion efforts. Collaboration among the Ministry of Health in Sudan, UNICEF and partners helped coordinate this social listening effort.

### 2.1 Developing gender-oriented messaging

Between September 2021 and April 2022 (one month after commencement of social listening by UNICEF), the Federal Ministry of Health launched three national gender-oriented social media campaigns in both the digital and interpersonal communication space in partnership with UNICEF. The initial social listening reports indicated that the three main categories of concern were (i) adverse events following immunization, (ii) questioning of vaccine efficacy, and (iii) gender-specific concerns. While the general public campaigns were refined to address category (i) and (ii) according to the social media findings, the gender-oriented campaign was developed to focus specifically on category (iii). The messages were directly informed by insights gathered through the social listening activities and phased in through the most popular social media platforms, being Facebook, Twitter, and Instagram accounts across the three campaigns.

The first gender-oriented campaign ran on social media between the 16th and 22nd September 2021, addressing four major gender-specific concerns around COVID-19 vaccines. The first message emphasized vaccine safety during pregnancy, asserting that there is no evidence that the vaccine adversely affects pregnancy. The second message reassured women that they can have healthy babies after having been vaccinated and stressed the fact that antibodies contained in the vaccine do not affect fertility. Other messages shared emphasized that the vaccine is safe to take during menstruation and there is no need to delay vaccination during this time or during lactation.

The second campaign was carried out from the 20th to 28th January 2022, reinforcing similar messaging through social media, and supplemented by home visits and community dialogues engaging face-to-face with approximately 270,000 individuals country wide. This effort was aided by the dissemination of 11,000 copies of factsheets with gender-oriented messaging (see Table 1).

**Table 1:** In-person reach of community engagement activities.

Timing	No. of community dialogues	No. of households	Home visits no. of HH individuals engaged
January, 2022	10,935	67,877	269,946
February–March, 2022	918	10,174	50,870
Total	11,853	78,051	320,816

Source: Data from UNICEF Sudan country office.

From the 19th February to 1st March 2022, the third gender-oriented campaign was launched on social media. This was accompanied by the hashtag “Says who?” and “Your story matters” and aimed to specifically encourage the public to question information received by word of mouth, helping to counter misinformation and rumors. Testimonies and advice from medical experts supplemented the third campaign. For instance, senior gynecologist Dr. Yagoub Mohammed Abdulmageed, was featured in an educational video that included gender-oriented messaging on the safety of COVID-19 vaccines (12). This video was broadcast both on national TV and radio as well as on the Federal and state MoH official Facebook pages. Additionally, 11,000 copies of women-centered fact sheets were developed and disseminated at gathering points and during home visits.

This stage of the campaign reinforced the need to check the reliability and source of key public health information. Stories were shared about common concerns amongst the community and what people found useful in overcoming them. The women-friendly “Says who?” campaign built on the culture of word of mouth that is common in Sudan, and also took into account evidence from the region which showed that people who were more sceptical of vaccinations tended to be more open to information from friends, families and neighbours than to expert sources of information (13). The campaign used innovative and attractive designs in vibrant colors, with content and captions in the local Sudanese Arabic dialect. They aimed to promote conversations and address rumors around COVID-19 vaccination, particularly in relation to fertility, breastfeeding, pregnancy, and menstruation. Moreover, about 52,000 community members across Sudan were engaged in face-to-face interpersonal communication approaches like home visits and community dialogue.

In addition to the above, UNICEF and the Ministry of Health conducted nationwide community engagement activities such as community dialogue by community leaders and home visits by trained health promoters—who were mostly female—to reach those less served by the social media campaign and to reinforce messages for those who may have limited internet access. Orientation sessions for women were also conducted at the health centers and in communities. See Table 1.

### 2.2 Data analysis

The Talkwalker App was programmed to provide a rapid snapshot of online engagement, disaggregated by sex before, during and after the gender-oriented campaign, led by the Federal Ministry of Health, UNICEF and partners. Talkwalker findings were triangulated with social media reports from the Federal Ministry of Health Facebook page as well as offline data. Offline data were gathered via weekly reports from UNICEF field offices covering all 18 states in Sudan. Qualitative analysis of the data revealed a range of concerns, rumours and misconceptions from communities, as well as sentiments, categorised under three broad themes, (i) adverse events following immunization, (ii) questioning of vaccine efficacy, and (iii) gender-specific concerns.

## 3 Results

Data from the Sudan dashboard on social listening (using the “Talkwalker” application) showed an overall 144% increase in the number of women engaged in COVID-19 related online conversation in the week following the launch of the first gender oriented campaign in September 2021. Talkwalker also continued to observe an overall increase in female engagement beyond the launch. The baseline measure in August 2021 showed 31% of engagement was from women, and 68% of engagement was from men. Female participation increased to 44% from September to October 2021 and reached its peak at 47% female in November 2021. Thereafter, online engagement figures continued to fluctuate between 35%–47% until February 2022. Overall, this reflects a sustained increase when compared to the baseline measure, before the gender-oriented campaigns. The three gender-focused COVID-19 campaigns had extensive social media reach, evident through the increase in reach figures from 4 million in December 2021 to 12 million by March 2022.

Analysis of the posts revealed that some posts were much more effective in driving the volume of interactions than others. Under the hashtags “Says who?” and “Your story matters”, seven social media posts attracted over 1,000 comments during the gender sensitive campaigns. These seven posts addressed variations on four themes—around vaccine safety during pregnancy, lactation and menstruation as well as that vaccines do not affect fertility among males and females alike.

**Figure 3:**
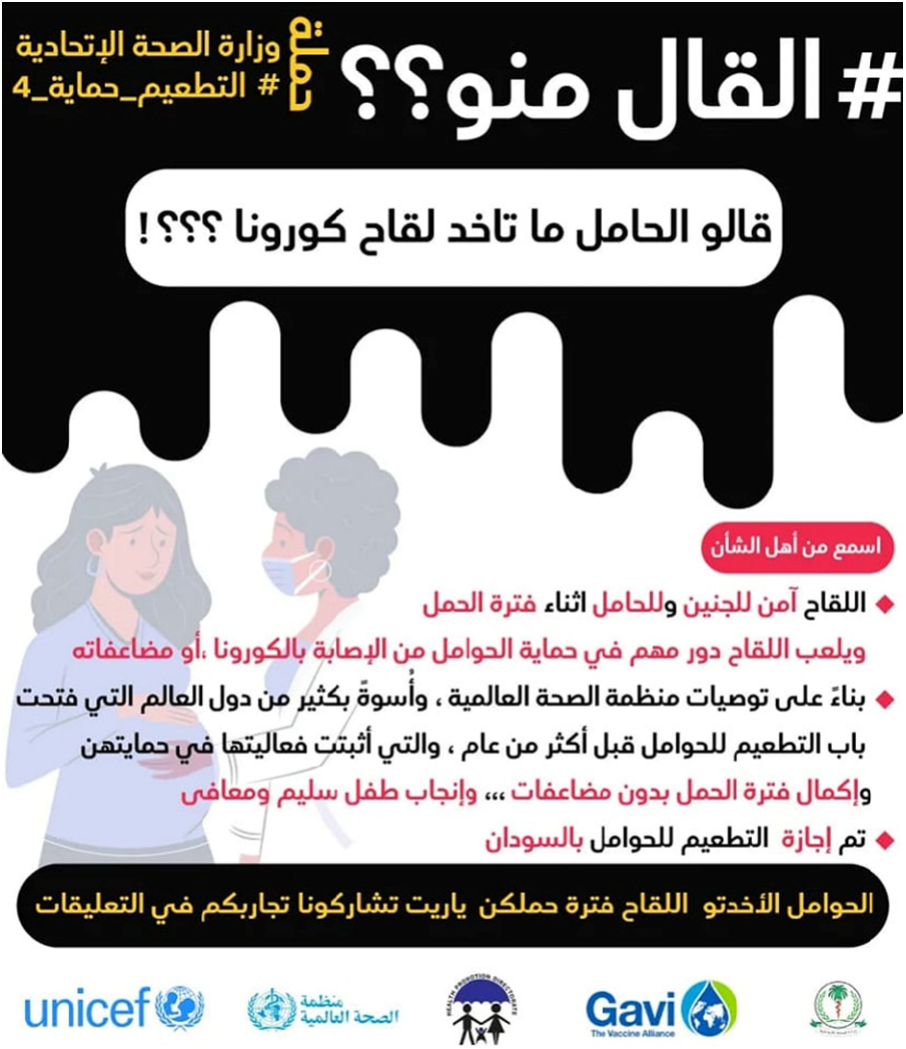
Social media example from the gender-oriented COVID-19 campaign, Sudan (Arabic).

These seven posts were broadly categorized into 4 major themes (i) women sharing positive experiences with COVID-19 vaccination during pregnancy and lactation, also reassuring other women that “*you and your baby will be safe after vaccination*”. For example, “*I took Janssen vaccine when my daughter was four months old, praise to God nothing happened to her, but I experienced pain at the injection site which lasted for 5 days*”. Another woman adds “*I was pregnant when I took the vaccine and now I gave birth and the baby and I are safe*”. Some women explained at length: “*I took Janssen vaccine a month ago, my daughter was 3 months old at the time and I breastfed her an hour following vaccination and nothing happened to her praise to God*”. The second theme related to the ability to conceive: “*My husband and I both got the two recommended vaccine shots and we are now expecting a baby*”, and “*I took the first dose before pregnancy and the second one during pregnancy and I gave birth safely*”. The third theme related to the availability of vaccines and the nearest vaccination sites, for example: “*Where can I get the second dose*” and “*Where can I find Pfizer booster dose in Khartoum*”. The fourth theme reflected a still hesitant group who wanted more reassurance from the health authorities as well as from other women engaged on-line. For instance, “*Can pregnant women get the dose*”, and “*Can lactating women take the shot*”. Figure 3 shows an example from the social media campaign. Table 2 provides examples of original posts in Arabic with English translation.

**Table 2:** Translation of selected posts from the gender-oriented COVID-19 campaign, Sudan.

Themes (original Arabic quote)	English Translation of social media posts
Theme 1. Sharing positive experiences	
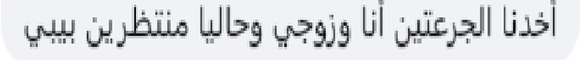	My husband and I both got the two recommended vaccine shots and we are now expecting a baby.
Theme 2: Ability to conceive	
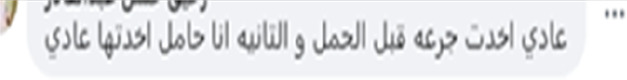	I took the first dose before preganacy and the second one during pregnancy and I gave birth safely.
Theme 3: Availability	
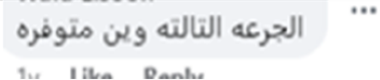	Where can I get the second dose?
Theme 4: Still hesitant	
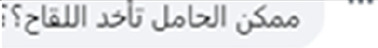	Can pregnant women get the dose?

Source: Talkwalker, UNICEF Sudan country office.

Social listening also revealed some unexpected consequences of vaccine misinformation. Some women reported taking the vaccine as a means of birth control, and some men also were avoiding the vaccine, fearing infertility. One comment claimed that one common reason motivating women to take the vaccine was because they did not want more children—under the false belief that the vaccine would prevent pregnancy. Further insights from social listening informed efforts to make vaccination centres more accessible and comfortable spaces for women. For example, women requested more female vaccinators and greater privacy. This rapid feedback and adaptation loop facilitated action by the Ministry of Health to address these concerns.

It is important to note that it is not possible to directly attribute uptake of vaccine to the intervention, as the effect of this intervention could not be isolated from other activities at the time, and longitudinal follow-up of social media participants was not feasible. However, the first and second gender oriented social media and in-person campaigns were associated temporally with a concomitant 9% increase in the national vaccination rate among women as illustrated in Table 3. Social listening platforms also have limitations in terms of capturing Arabic language and its many colloquial forms and expressions. In addition, digital system constraints hindered the availability of gender segregated data on vaccination uptake beyond this period and made it difficult to isolate any possible impact of the third gender oriented campaign on women's vaccine uptake.

**Table 3:** COVID-19 total vaccination doses administered in Sudan between 9th march, 2021 to 23rd January, 2022 segregated by sex.

Vaccine round	Time frame	Female	Male	Total
1	9 March–11 July 2021	374,015 (31.9%)	799,357 (68.1%)	1,173,372
2	29 Aug 2021–23 Jan 2022	246,524 (39.7%)	374,696 (60.3%)	621,220
**Total**		**620,539**	**1,174,053**	**1,794,592**

Source: Federal Ministry of Health in Sudan.

## 4 Discussion

The speed at which COVID-19 spread overwhelmed health systems initially. Although disaggregation of data by important socio-demographic features, such as sex and age, is essential in epidemiology and public health, it took time for this data to be available during the pandemic. In addition, most countries did not have existing systems and processes for monitoring and building on social and behavioral data. As such, social listening presents itself as a very useful tool for immediate, “24/7” real-time scanning of sentiments and misinformation despite the well-documented limitations of social media in terms of representativeness (14).

Given these benefits, social listening is recommended for a range of purposes, as an adjunct to traditional data collection. The ultimate value of social listening for gender equality, or any broader programmatic interventions, is that it can enhance risk-informed design and the ability to adapt programmes and campaigns in real time. As an approach, social listening can be replicated and scaled up for community as well as service-related issues, identifying community priorities, fostering inclusion, and ultimately improving service uptake of humanitarian and development interventions. Social listening insights also have the potential to increase programme effectiveness and hence value for money. As a rapid and user-friendly tool, social listening is also useful in emergency responses as a two-way community feedback tool to facilitate risk communication and community engagement—especially when other means of engagement may be difficult, as was the case during the COVID-19 pandemic. Social listening also holds great potential to inform more responsive development programming by identifying trends in real time, on topics related to social norms related topics.

In Sudan, social listening both validated suggested concerns and revealed new and nuanced concerns related to women's uptake of the COVID-19 vaccine that may have otherwise been missed. Intriguingly, our data showed a dichotomy where some women avoided the vaccine due to fears of infertility, while others used it as a de facto means to prevent unwanted pregnancy because of the same misinformation around infertility.

In this context, two key innovations were the rapid real-time monitoring of public attitudes and the large-scale tracking of sentiment data through social media, complemented by off-line feedback. In countries like Sudan, tracking and assessing data from the state to the federal level can be time-consuming and opportunities for timely adaptations or improvements may be missed. Social listening enabled the campaign to provide tailored and gender-sensitive information to women. Without social media, this scale of coverage and feedback generation would be prohibitively resource intensive, and likely too slow to keep pace with the changing sentiments of the community.

More broadly, it is recommended that social listening is utilised not only passively, but also proactively. For example, passive application is the most common whereby social listening platforms are used to gather and organize existing public attitudes towards key health behaviors or to monitor what public health information is being shared (15, 16). Proactive application can include approaches such as utilizing social listening data systematically as part of formal programming monitoring of planned interventions which may allow inferences based on observations of volume and type of posts, including sentiment analysis. These approaches can help to mitigate misinformation and make adjustments based on public sentiment that can shape more relevant and effective public health action. Frameworks and guidance, especially for low- and middle-income countries that do not have existing social listening systems in place, should therefore be continually developed and deployed (17), especially in countries affected by instability and conflict where other data collection methods may be difficult.

While our study illuminates the potential benefits of social listening and gender-responsive messaging in promoting vaccine equity, it is not possible to directly link the observed increase in vaccine uptake to our intervention. Realistically, the synergistic effect of combining several strategies with social listening is more likely to achieve results than any single strategy in isolation, especially in an emergency. As such, effective integration of offline and online engagement, coupled with a consistent level of rigour and systematic tracking and evaluation will be important to future success. Unfortunately, basic disaggregation of data is still not routine in many low resource settings. As such, disaggregation by variables such as gender, age, education, disability, and ethnicity needs to be specifically requested, to offer deeper insights into the impact of these programs. Equally, expanding access to online channels will also improve the value of social listening.

Importantly, in this case, social listening informed gender-responsive messaging, provided a space for women's concerns to be voiced, addressed low vaccine acceptance, and resulted in enhanced female engagement on social media. Crucially, the questions and fears were shared in close-to-real time, and authorities were able to respond to this feedback, thus increasing trust and confidence in the services. Social listening has the potential to provide invaluable insights into other dimensions of people's lives, addressing their multiple roles, broader gender norms and harmful practices—beyond public health concerns alone, which is particularly relevant for emergency situations.

These findings demonstrate the value of integrating real-time feedback with large-scale social media campaigns to refine interventions, particularly in a conflict-prone and low-income context. Our experience reinforces the notion that “one size does not fit all” when it comes to health-related communication. Responses should be tailored, contextualized, and person-centered, particularly in addressing concerns unique to women.

## Data Availability

The raw data supporting the conclusions of this article will be made available by the authors, without undue reservation.
